# Individual Precursors of Student Homework Behavioral Engagement: The Role of Intrinsic Motivation, Perceived Homework Utility and Homework Attitude

**DOI:** 10.3389/fpsyg.2019.00941

**Published:** 2019-04-26

**Authors:** Natalia Suárez, Bibiana Regueiro, Iris Estévez, María del Mar Ferradás, M. Adelina Guisande, Susana Rodríguez

**Affiliations:** ^1^Department of Psychology, University of Oviedo, Oviedo, Spain; ^2^Department of Psychology, University of A Coruña, A Coruña, Spain; ^3^Department of Pedagogy and Didactics, University of A Coruña, A Coruña, Spain; ^4^Department of Developmental and Educational Psychology, University of Santiago de Compostela, Santiago de Compostela, Spain

**Keywords:** homework, behavioral engagement, intrinsic motivation, perceived utility, attitude, secondary education

## Abstract

Currently, the concept of *engagement* is crucial in the field of learning and school achievement. It is a multidimensional concept (e.g., behavioral, emotional, and cognitive dimensions) that has been widely used as a theoretical framework to explain the processes of school engagement and dropout. However, this conceptual framework has been scarcely used in the field of homework. The aim of the present study was to analyze the role of intrinsic motivation, perceived homework utility, and personal homework attitude as precursors of student homework engagement (behavioral engagement) and, at the same time, how such engagement is the precursor of academic achievement. Seven hundred and thirty students of Compulsory Secondary Education (CSE) (7th to 10th grade) from fourteen schools northern Spain participated. A structural equation model was elaborated on which intrinsic motivation, perceived utility and attitude were observed variables, and student engagement (time spent on homework, time management, and amount of teacher-assigned homework done) and academic achievement (Mathematics, Spanish Language, English Language, and Social Science) were latent variables. The results reveal that (i) intrinsic motivation is a powerful precursor of student behavioral engagement (also perceived utility and attitude, although to a lesser extent), and (ii) academic achievement is closely linked to the level of student engagement, qualifying the results of many of the previous studies conducted from a task-centered perspective (as opposed to a person-centered perspective).

## Introduction

In accordance with the proposal of Trautwein et al. (2006), we study herein the role of motivational variables as individual antecedents of student behavioral homework engagement and its impact on academic achievement. Assuming the principles of the theory of expectancy-value (Eccles, 1983; Pintrich and De Groot, 1990; Eccles and Wigfield, 2002), we focused this study on the role of the motivational variables related to the value attributed to homework and we addressed the construct of engagement in accordance with the contributions of the theory of school engagement (Fredricks et al., 2004).

Nowadays, it seems little debatable that the value attributed by students to academic tasks such as tests or homework is linked to their engagement and the effort dedicated to these tasks (Greene et al., 2004; Xu, 2005; Cole et al., 2008). Thus, students with high-value beliefs spend more time, devote more effort, and complete more homework than those who do not value academic activity (Bong, 2001; Miller and Brickman, 2004; Wise and DeMars, 2005; Liem et al., 2008; Eccles and Wang, 2012). This attributed value thereby indirectly influences their achievement (Pintrich and De Groot, 1990; Wolters and Pintrich, 1998; Wigfield and Eccles, 2002; Trautwein et al., 2006).

### Motivation and Homework Behavioral Engagement

Compared with students who do their homework to avoid blame or to please their parents, the evidence suggests that intrinsically motivated students devote more effort, persist more, and obtain better results when they engage in an activity (Wigfield and Eccles, 2002; Hardre and Reeve, 2003; Coutts, 2004; see the review of Wigfield et al., 2009). Along with personal expectancies, the link between the value attributed to homework and the intentions of learning and devoting effort is well documented in the literature (Bandura, 1997; Wigfield and Eccles, 2000; Eccles and Wigfield, 2002; Wigfield et al., 2009; Metallidou and Vlachou, 2010). Assuming the principles of the theory of Expectancy-Value, this study aims at verifying to what extent the value students attribute to homework predicts their intentions and real decision to engage in homework and to do it (Eccles et al., 1993; Eccles, 2005; Wigfield et al., 2017).

Most of the research that supports the expectancy value models has argued that the value attributed to homework has at least three dimensions or components: the degree to which it is perceived as interesting—its intrinsic value— personally significant and important for the student—achievement value—, and useful—utility value. Thus, students who consider homework important, useful, and/or interesting hold high self-efficacy beliefs and persevere in the face of difficulties encountered when doing homework (Bandura, 1997). In fact, this value-effort relationship has been found for homework, showing the direct influence of the value attributed to dedication and engagement (Trautwein et al., 2006; Hong et al., 2009; Xu, 2017; Xu et al., 2017), and underlining the importance of the utility perception of homework in the promotion of diverse academic outcomes (Trautwein et al., 2006; Yang et al., 2016; Xu et al., 2017). The term attitude is understood as an evaluative predisposition (positive or negative) that conditions the subject to perceive and to react in a determined way in light of the objects (people, groups, ideas, situations, etc.). It is a learned predisposition, not innate, and stable although it can change (Hidalgo et al., 2004). Therefore, the attitude toward homework refers to the positive or negative predisposition of these students to do homework.

### Homework Behavioral Engagement and Academic Achievement

School engagement is receiving increasingly more attention in psychological research because it has been shown to be a relevant predictor of different educational outcomes (Ladd and Dinella, 2009; Wang and Peck, 2013), and specifically, of academic achievement (Ladd and Dinella, 2009; Reeve and Tseng, 2011). Although there are significant variations in the implementation of the construct, we consider engagement as a meta-construct with affective-emotional, cognitive, and behavioral subcomponents (Fredricks et al., 2016; Rodríguez-Pereiro et al., 2019).

In this context, the review of students’ behavioral engagement usually refers to their participation at school, indicators of pro-social behavior in academic contexts, compliance with rules, and/or dedication to homework (e.g., Fredricks et al., 2004; Christenson et al., 2012). Behavioral engagement, in terms of time, effort, amount of homework performed, persistence, and/or dedication (Eccles and Wang, 2012), must have an impact on adolescents’ academic achievement (King, 2015; Mikami et al., 2017).

The construct student homework behavioral engagement usually includes behavioral indicators concerning the time devoted to homework, the management of that time, or the amount of homework performed (Trautwein et al., 2006).

Although among other factors, achievement could depend on students’ age, the quality of the assigned homework, and/or the procedure used to measure achievement, research tends to support a positive relationship between the amount of homework carried out and academic achievement (e.g., Cooper et al., 1998, 2006; Cooper and Valentine, 2001; Epstein and Van Voorhis, 2001; Trautwein et al., 2002; Fernández-Alonso et al., 2015; Núñez et al., 2015a).

Some works have found positive relationships (see review of Cooper, 1989; Cooper and Valentine, 2001; Cooper et al., 2006; Fernández-Alonso et al., 2015), with more obvious effects in secondary education than in primary education, and some studies have shown that the time spent on homework and achievement may not be related or may even be negatively related (De Jong et al., 2000; Trautwein, 2007; Kitsantas et al., 2011). There may be a differential effect of the time devoted to homework, and also of the amount of homework performed, at the classroom and individual level.

Both students’ committed effort and their good use of homework time have a positive effect on their achievement (Schmitz and Skinner, 1993; Trautwein and Köller, 2003; Trautwein et al., 2006; Xu, 2013). In this sense, Xu (2010) concluded, for example, that a good study time management contributes to completing a greater amount of homework. Trautwein (2007) found that effort is a better predictor of achievement than time spent on homework. As proposed by Núñez et al. (2015a), the use of homework time could positively affect academic achievement insofar as it contributes to increasing the amount of homework performed.

### The Present Study

According to Lawson (2017), behavioral engagement is a manifestation of internal motivational processes such as intrinsic motivation, self-efficacy, or the value attributed to homework (Becker et al., 2010; Schiefele et al., 2012; Guthrie et al., 2013), which energize and direct action. In this study, we focus on the value component in terms of the conceptual model of homework developed by Trautwein and colleagues and tested in various studies (e.g., Trautwein and Lüdtke, 2007; Dettmers et al., 2010, among others). As in other studies of this field (Hughes et al., 2008; King, 2015; Mikami et al., 2017), we propose a structural model in which *homework behavioral engagement* (i.e., the amount of time dedicated to doing teacher-assigned homework; homework time management; and the amount of homework assigned) *mediates* between certain student motivational conditions—*students’ motivational conditions* (perceived homework utility; homework intrinsic motivation; and homework attitude) and their *general academic achievement* (Social Sciences, Math, Language, and English as second language). In the present study we focus on students in grades 7–10, it is the proper age in which they should begin to take importance the accomplishment of homework. Despite the large number of research on homework in secondary education, it seems interesting to begin to verify models of relationships that allow us to interpret adequately the relationships between motivation and behavioral engagement.

**FIGURE 1:**
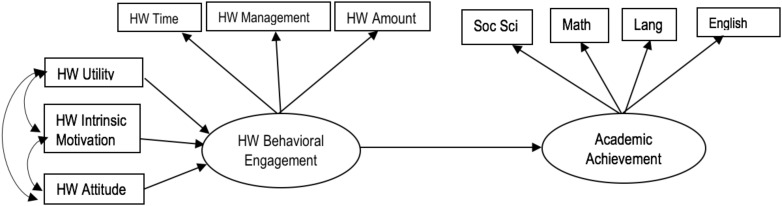
Structural model to be tested.

Figure 1 shows the model to be tested. The main hypotheses of this model are as follows:

(1) Students’ homework behavioral engagement will be significantly and positively determined by their motivational conditions (homework intrinsic motivation, homework utility, and homework attitude). Based on previous studies (e.g., Trautwein et al., 2006; Hong et al., 2009; Regueiro et al., 2015, 2017, 2018; Valle et al., 2018; Yang et al., 2016; Xu, 2017; Xu et al., 2017), we expect that the intensity of this relationship (in terms of the effect size) will be medium or large.
(2) Students’ homework behavioral engagement will positively and significant predict their overall academic achievement (in terms of average grades in the four core academic areas). Based on the results of previous studies of the relationship between homework and academic achievement in Secondary Education students (e.g., De Jong et al., 2000; Trautwein et al., 2002; Cooper et al., 2006; Trautwein, 2007; Kitsantas et al., 2011; Fernández-Alonso et al., 2015; Núñez et al., 2015a; Fan et al., 2017), we expect that the effect size of the relationship will be moderate (or small).

## Materials and Methods

### Participants

Participants were 730 students in Compulsory Secondary Education (CSE) (aged between 12 and 16 years (*M* = 13.5, *SD* = 1.15) from 14 schools randomly selected (12 public schools and 2 private-subsidized schools) in three provinces of northern Spain. Fifty-six students were eliminated due to missing data. Half of the schools are in urban areas and the other half are in rural or semi-urban areas. Of the participants, 43.4% were boys and 56.6% were girls. Besides, 194 students (26.6%) were in 1st grade of CSE, 152 students (20.8%) were 2nd-graders, 182 students (24.9%) were in 3rd grade, and 202 students (27.7%) were 4th-graders.

### Instruments

#### Student’s Motivational Variables

The items used to measure homework intrinsic motivation, homework perceived utility, and homework attitude were obtained from the Homework Survey, an instrument already used in previous studies (e.g., Núñez et al., 2015a,b,c; Valle et al., 2015a, 2018). The fact of having chosen the questionnaire as a data collection instrument was mainly due to its characteristics of versatility, efficiency and generalizability, which have made this research instrument one of the most widespread in the educational and psychological field, as established authors such as McMillan and Schumacher (2005).

*- HW Intrinsic Motivation*. We evaluated the students’ degree of enjoyment, satisfaction, and the benefits obtained by doing homework. This dimension consists of 8 items (α = 00.85), which are rated on a 5-point Likert-type scale ranging from 1 (*completely false*) to 5 (*completely true*). An example item is: “I enjoy doing homework, because it allows me to learn more.”

*- HW Perceived Utility*. This variable was assessed with a single item asking students whether they considered the homework assigned by their teachers to be useful. The response scale ranged from 1 (*completely false*) to 5 (*completely true*).

*- Homework Attitude*. In this study three items to evaluate the affective dimension of the homework attitude were used: students’ preference for, their willingness to (their disposal to), and their positive emotions generated and associated with doing homework (α = 0.77). Students responded on a 5-point Likert-type scale ranging from 1 (*completely false*) up to 5 (*completely true*).

#### Homework Behavioral Engagement

Behavioral engagement was measured through three indicators: time spent on homework, homework time optimization, and amount of teacher-assigned homework carried out by the students. The items used to obtain three measurements were taken from the aforementioned Homework Survey.

*- Homework Time Spent*. To measure the time spent on homework, students responded to two items (“How much time do you usually spend on homework every day from Monday to Friday?,” and “How much time do you usually spend on homework on the weekend?), with the following response options: 1 (*less than 30 min*), 2 (*30 min to 1 h*), 3 (*1 h to an hour and a half*), 4 (*1 h and a half to 2 h*), and 5 (*more than 2 h*). The alpha coefficient was α = 0.72 in this study).

*- Homework Time Management*. This variable was measured through the responses to two items asking students to indicate how they managed the time normally spent doing homework (Monday through Friday, and on the weekend), using the following scale: 1 (*I waste it completely; I am constantly distracted by anything*), 2 (*I waste it more than I should*), 3 (*regular*), 4 (*I manage it pretty well*), and 5 (*I optimize it completely; I concentrate and, I don’t think about anything else until I finish*). The alpha coefficient was α = 0.78 in this study.

*- Amount of Homework Done.* The estimate of the amount of teacher-assigned homework completed by students was obtained through one item rated on a 5-point Likert-type scale: 1 (*none*), 2 (*some*), 3 (*one half*), 4 (*almost all*), and 5 (*all of it*).

#### Academic Achievement

The evaluation of academic achievement was calculated from average grade obtained by the students at the end of the academic year they were enrolled in at that time. The subjects used to calculate the mean were Social Sciences, Mathematics, Spanish Language, and Foreign Language (English as a second language) because they have the greatest weight in the curriculum.

### Procedure

The data referring to the variables under study were collected during school hours by personnel external to the school itself, after obtaining the written informed consent of the parents or legal guardians, the management team, and the students’ teachers, respecting the ethical standards established in the Declaration of Helsinki. In each session, the staff give some practical indications to students on how to address those questions. Then, participants fill in all the questions of the self-report individually by themselves, and without time limit.

### Data Analysis

After verifying that the distribution of the variables could be considered sufficiently normal to allow the use of the maximum likelihood procedure, a structural equation analysis, using the computer program AMOS 18, was employed to contrast a hypothesized model predicting the influence of homework motivation on homework engagement and achievement. In addition to chi-square (χχ^2^) and its associated probability (*p*), we used two absolute indices: the goodness-of-fit-index (GFI) and the adjusted goodness-of-fit-index (AGFI). We also provide a relative index, the comparative fit index (CFI) (Bentler, 1990); and a close-fit parsimony-based index, the root mean square error of approximation (RMSEA), including 90% confidence intervals (Hu and Bentler, 1999). The model fits well if GFI and AGFI >0.90, CFI >0.95, and RMSEA ≤ 0.05.

The effect sizes were calculated using Cohen’s *d* (*d* < 0.20 = non-significant effect; *d* ≥ 0.20 and *d* < 0.50 = small effect; *d* ≥ 0.50 and *d* < 0.80 = medium effect; *d* ≥ 0.80 = large effect).

## Results

### Preliminary Analysis

Table 1 shows the means, standard deviations, skewness, kurtosis, and bivariate Pearson correlations. In general, the relationship between the variables included in the study was as expected. Specifically, the three motivational variables considered— intrinsic motivation, utility, and homework attitude—significant and positive correlations with the time spent doing homework, time optimization, and the amount of homework done. These three variables that constitute the construct of homework behavioral engagement correlated positively and significantly with each other and with the grades obtained by the students in the four subject areas considered.

**Table 1:** Descriptive statistics and Pearson correlations (*N* = 730).

	1	2	3	4	5	6	7	8	9	10
1. HWUT	—									
2. HWIM	0.612**	—								
3. HWAT	0.459**	0.520**	—							
4. HWBE_1	0.266**	0.227**	0.174**	—						
5. HWBE_2	0.390**	0.450**	0.321**	0.397**	—					
6. HWBE_3	0.331**	0.381**	0.337**	0.193**	0.396**	—				
7. AAch_1	0.137**	0.221**	0.089*	0.183**	0.327**	0.221**	—			
8. AAch_2	0.119**	0.167**	0.104	0.172**	0.313**	0.156**	0.664**	—		
9. AAch_3	0.149**	0.224**	0.096	0.171**	0.304**	0.159**	0.807**	0.691**	—	
10. AAch_4	0.119**	0.198**	0.066	0.119**	0.297**	0.161**	0.715**	0.667**	0.751**	—
*M*	3.49	3.51	2.11	3.03	3.97	3.23	6.42	5.64	6.06	5.93
*SD*	1.074	0.793	0.863	1.151	1.119	1.066	2.283	2.325	2.111	2.385
Skewness	-0.517	-0.523	0.667	0.014	-0.922	-0.247	-0.304	-0.139	0.032	-0.109
Kurtosis	-0.289	-0.004	-0.105	-0.821	-0.229	-0.495	-0.468	-0.639	-0.615	-0.743


*HWUT, Homework Utility; HWIM, Homework Intrinsic Motivation; HWAT, Homework Attitude; HWBE_1, Homework Behavioral Engagement: Time Spent; HWBE_2, Homework Behavioral Engagement: Amount of Teacher-assigned Homework Done; HWBE_3, Homework Behavioral Engagement: Homework Time Management; AAch_1, Social Science Achievement; AAch_2, Mathematics Achievement; AAch_3, Language Achievement; AAch_4, English Achievement as Second Language. ^∗∗^*p* < 0.01. ^∗^*p* < 0.05.*

We observed moderate correlations between the utility perception and the intrinsic value of homework and students’ grades, whereas the interrelationship between homework attitude and academic achievement was lower. Statistically significant correlations were also observed among the three homework motivational variables, as well as among the grades obtained in the subjects that constitute the academic achievement measures.

### Structural Model Fit

In Figure 1, the relationships expressed in the formulation of the hypothesis of the contrasted model are made explicit. With the exception of χ^2^(31) = 75.548; χ^2^/*df* = 2.43, *p* < 0.001, all the fit indices suggest that the hypothesized model adequately represents the relations of the empirical data matrix: GFI = 0.980; AGFI = 0.964; TLI = 0.980; CFI = 0.986; and RMSEA = 0.044, 90% CI [0.032, 0.057], *p* > 0.05. As a result, the model does not need any changes. In addition, as can be seen in Table 2, the factor loadings as well as the corresponding estimation errors of the three measurement variables corresponding to student homework behavioral engagement (time spent; homework time management; amount of homework done) and to the academic achievement areas (Social Sciences, Mathematics, Spanish Language, and English as Second Language) suggest that both latent variables were reliably constructed.

**Table 2:** Assessment of the hypothesized homework model.

	SRW	SE	CR	*p*	*d*
**Structural Model**					
HW Utility → HW Behavioral Engagement	0.212	0.039	4.397	0.000	0.330
HW Attitude → HW Behavioral Engagement	0.117	0.045	2.628	0.009	0.195
HW I. Motivation → HW Behavioral Engagement	0.414	0.056	8.125	0.000	0.631
HW Behavioral Engagement → Academic Achievement	0.418	0.099	8.696	0.000	0.680
HW I. Motivation ↔ HW Attitude	0.345	0.028	12.423	0.000	1.035
HW I. Motivation ↔ HW Utility	0.517	0.037	14.052	0.000	1.218
HW Attitude ↔ HW Utility	0.424	0.038	11.267	0.000	0.918
**Measurement Model**					
HW Behavioral Engagement → HW Time Spent	0.475	0.060	10.463	0.000	0.840
HW Behavioral Engagement → HW Time Management	0.534	0.057	11.499	0.000	0.941
HW Behavioral Engagement → Amount HW Done	0.776	—	—	—	—
Academic Achievement → Social Sciences	0.877	0.044	25.635	0.000	6.007
Academic Achievement → Mathematics	0.772	—	—	—	—
Academic Achievement → Spanish Language	0.909	0.040	26.633	0.000	11.712
Academic Achievement → Second Language (English)	0.829	0.046	23.986	0.000	3.857


*HW, Homework; SRW, Standardized Regression Weights; SE, Standard Errors; CR, Critical Ratio; *p*, Probability; *d*, Effect Size.*

### Assessment of Model Hypotheses

**FIGURE 2:**
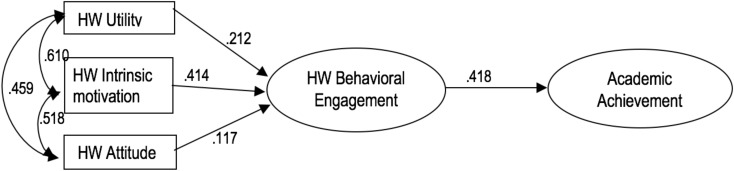
Correlations and standardized regression weights for the final model. All coefficients are statistically significant at *p* < 0.001, except for HW Attitude on HW Behavioral Engagement (*p* < 0.01).

Correlations between the three independent variables, standardized regression weights, and their statistical significance are presented in Table 2 and Figure 2.

In the present study, two general hypotheses were formulated. First, we hypothesized that students’ homework behavioral engagement would be significantly and positively determined by their motivational personal variables. In addition, based on previous studies, we expected that the intensity of this relationship would be medium or large. In general terms, the results confirm this hypothesis. As a whole, the effect is statistically significant and positive: students who perceive greater homework utility have a more positive attitude toward homework and consider it an opportunity to learn. They also engage more in their homework than students who express low utility, a poor attitude, and low intrinsic motivation. However, the effect sizes suggest that students’ homework behavioral engagement depends little on perceived homework utility and homework attitude, although it does depend on intrinsic homework motivation (interest in working on homework to achieve learning and gain competence), with an effect size between medium and large. The three motivational variables explain 17.5% of students’ homework behavioral engagement.

Secondly, we formulated the hypothesis that students’ homework behavioral engagement would significantly and positively predict their overall academic achievement, and that the effect size of that relationship would be moderate, or even small. The data obtained confirm this hypothesis, both in the intensity (the mean effect size) and the sign (positive). The higher the students’ homework behavioral engagement, the greater was their academic achievement, and vice versa. The amount of total explained academic achievement variance was 41%.

## Discussion

The role of students’ behavioral homework engagement is a highly controversial issue. For example, prior studies indicate that spending more time on homework is no guarantee of higher academic achievement. Also, there is not sufficient empirical evidence about the determinants of such engagement. This research intended to provide some information about these two large gaps. On the one hand, we wondered whether the motivational factors could be important determinants of student homework engagement (as derived from the motivational theories of academic learning) and, on the other hand, we wished to confirm the predictive power of student homework engagement for academic achievement when using latent variables (instead of specific measures of engagement or achievement).

The results confirm the contribution of motivation and, specifically, of its value component, on students’ academic engagement (Bong, 2001; Eccles and Wang, 2012). Moreover, according to our results, the value attributed to homework in terms of enjoyment and satisfaction, utility perception, and positive attitude moderately explain students’ dedication to and engagement with homework.

Specifically, when students approach homework due to their interest, in order to learn and acquire competence, they spend more time, optimize the time spent, and also do more homework (Trautwein et al., 2006; Hong et al., 2009; Xu et al., 2017). As defended from different theoretical frameworks, interest would contribute to achievement to the extent that, in general, it increases behavioral engagement, dedication, management of the learning process, and the attentional resources that are implemented (Lee et al., 2014; Trautwein et al., 2015; Harackiewicz et al., 2016). The prescription and correction of homework can become an instructional strategy for the learning promotion and academic performance, as teachers manage to adjust to the needs and interests of their students (e.g., Akioka and Gilmore, 2013). Beyond the interventions focused on self-monitoring and self-management (e.g., Breaux et al., 2019) or the use of reinforcements (Reinhardt et al., 2009), homework that are prescribed from classroom must be meaningful and purposeful if we want the apprentices to actively engage with them (Kalchman and Marentette, 2012).

Likewise, it seems that homework utility perception contributes somewhat to helping students spend more time on homework, better manage that time, and do more homework (Cooper et al., 2006; Yang et al., 2016; Fan et al., 2017). Intrinsic motivation and perceived utility also guarantee a more positive attitude toward doing homework. Given the strong association found, if students perceive the utility of the assigned homework, they could improve their more intrinsic reasons for engaging in homework, which would promote more positive attitudes toward such engagement.

The value students attribute to homework, a key aspect of motivation in self-regulated learning models (Pintrich and Zusho, 2007; Wigfield and Cambria, 2010), should be understood as a multidimensional construct that integrates students’ personal interests and the interest aroused by the situations, but also their estimates of its importance or usefulness. As learners will probably engage intrinsically in their homework if they perceive its utility, and in view of the fact that direct intervention in the intrinsic value of homework is not always easy and could even undermine students’ sense of autonomy (Deci and Ryan, 1985), homework utility value becomes a core support in the educational intervention with students who show little interest in homework.

Thus, as Epstein and Van Voorhis (2001, 2012) concluded, when teachers explicitly present the meaning and utility of the homework they assign, they could be affecting students’ behavioral engagement and homework time management. In general, the research seems consistent, suggesting that student homework engagement could be optimized if the teacher assigns quality homework, that is, homework perceived as useful and interesting, which enables students’ progress (adapted to the potential of each student or group of students) and is causally linked to academic success (e.g., Trautwein et al., 2006; Trautwein and Lüdtke, 2009; Dettmers et al., 2010, 2011;Rosário et al., 2018).

In any case, we should not lose sight of the fact that the explanatory potential of the motivational variables considered herein is relatively low and, in fact, more than 80% of the variability of homework behavioral engagement would be explained by variables that were not included in this work. In this regard, we acknowledge that we did not address the expectancy component of motivation, which, as defended from different theoretical frameworks (Eccles, 1983; Pintrich and De Groot, 1990; Bandura, 1997; Eccles and Wigfield, 2002), can be considered a predictor of homework behavioral engagement, at least in terms of effort and persistence (Trautwein et al., 2006; Nagengast et al., 2013). On the other hand, although we must assume that motivation energizes cognitive engagement (Greene et al., 2004; Greene, 2015), in this case, we did not study the resources and learning strategies implemented by students when approaching homework. However, the research of Valle et al. (2015b) allows us to hypothesize the importance of intrinsic motivation and attitude in the decision to engage more or less deeply in homework, and thereby related to homework behavioral engagement.

On another hand, as has already been stated by many previous studies (Cooper et al., 1998, 2006; Cooper and Valentine, 2001; Epstein and Van Voorhis, 2001; Trautwein et al., 2002; Xu, 2010; Fernández-Alonso et al., 2015; Núñez et al., 2015a), the time spent on homework along with good time management the amount of homework done largely contribute to students’ grades in different curricular subjects. Compared with other studies that found null or negative relationships (e.g., see De Jong et al., 2000; Trautwein, 2007; Kitsantas et al., 2011), the results of this research not only corroborate the positive relationship between behavioral engagement measures and academic achievement, but also show that the effect size is higher than that reported in most of the previous studies. High school students who spend more time, manage that time well, and do all the homework clearly perform better than those who dedicate little time, are easily distracted, or do not finish their homework.

If, indeed, the more students engage in their homework, the better grades they obtain, then doing homework is better than not doing homework, and assigning homework in class will therefore contribute to improving students’ academic achievement. In this regard, no doubt, students’ competence and abilities will mediate their management of resources like time, the environment, or help (Du et al., 2016; Xu et al., 2017), as well as the role of parents, teachers, and peers (Núñez et al., 2015b,c).

Finally, as student engagement and dedication to homework impact on their academic results and depend to some extent on homework utility perception, parents and teachers need to converge so we can sustain the utility perception of homework as a society. In this sense, there is a risk that the increasing and recurrent loss of prestige of homework will end up diminishing students’ intrinsic motivation and promoting a negative attitude toward homework.

### Limitations of the Work and Future Research

Although the results of the study seem to be robust (consistent effects of the predictions, estimation errors within normal parameters, etc.), they should be taken with some precaution due to some limitations inherent in the nature of the data of the study, the sample used, or the measuring instruments.

The research is cross-sectional, so any causal inferences are seriously compromised. Although we used a powerful multivariate strategy to analyze the data, which could lead us to think in terms of causality, this is not possible because, for this purpose, we should have used a longitudinal design (three repeated measures could be sufficient for this model) or an experimental design. Although in the present investigation, we chose a cross-sectional strategy, we accept and appreciate the suggestion of Xu et al. (2017) about the need to develop causal research where the effects of homework assignment—type of tasks, frequency, etc.—and teacher feedback on students’ motivation and homework engagement are confirmed. In line with different works of research within the framework of the expectancy-value models (e.g., Durik et al., 2006; Simpkins et al., 2006), it also seems interesting to begin to develop longitudinal follow-up studies that allow us to determine whether, indeed, students’ attitudes and motivation have a greater explanatory potential for homework behavioral engagement throughout their schooling and to observe the extent to which we can assume evolutionary changes in the influence of homework on academic achievement.

Another limitation has to do with the student sample used in this study. We must admit that the results could vary significantly if the sample had been obtained randomly and were representative of the population from which it comes (educational stage, types of educational centers, sociometric features of the families, etc.). However, we are confident that the procedure used is sufficiently sensitive to the variables and that it has strengthened the reliability of the results described.

Finally, data collection regarding homework was done through self-reports. Although this methodology is commonly used in psychology and education, possibly essential to measure thoughts and behaviors that are otherwise hardly observable, it is necessary to replicate the findings using complementary strategies and measuring instruments (of various types). In addition, some variables of this study were assessed with a relatively low number of items, which may compromise the robustness of these measures (although consistency coefficients higher than 0.70 are usually considered reliable). In relation to this type of measure, a matter which we must not forget when interpreting the data and drawing conclusions and implications for educational practice, is that the information obtained is self-reported, which may be more or less subjective, depending on the individual’s variables and the variables of the context. For example, homework utility in itself was not considered, but instead students’ utility perception. Reality and perception of reality may not coincide completely.

Finally, we emphasize that, in this investigation, like in many others carried out within the field of education, we used students’ grades at the end of course as an indicator of academic achievement. However, it should not be forgotten that the magnitude of the relationship between student homework engagement and academic achievement could be significantly different if we had used a more objective measure of achievement (for example, the result of a standardized achievement test). Nevertheless, this study used the final grades as a measure of achievement due to its markedly ecological nature (compared to the standardized test).

This work allows us to suggest the need to incorporate motivational variables such as interest, usefulness and attitude toward homework in research agendas given the incidence found for active participation and student dedication. It is also important to emphasize the need to develop improvement programs, integrated into the school curriculum and implemented from schools with the involvement of parents.

## Ethics Statement

Does the study presented in the manuscript involve human or animal subjects: Yes.

This study was carried out in accordance with the recommendations of Research and Teaching Ethics Committee of the University of A Coruña and the Declaration of Helsinki. The protocol was approved by the Research and Teaching Ethics Committee of the University of A Coruña and the Declaration of Helsinki.

Data about the target variable were collected in accordance with the recommendations of the ethical standards established in the Research and Teaching Ethics Committee of the University of A Coruña and the Declaration of Helsinki. This study was carried out with the written informed consent from parents or legal guardians.

## Author Contributions

NS, BR, and IE contributed to conception and design of the study. MdMF organized the database. MG and SR performed the statistical analysis. NS, BR, and IE wrote the first draft of the manuscript. MdMF, MG, and SR wrote the sections of the manuscript. All authors contributed to manuscript revision, read and approved the submitted version.

## Conflict of Interest Statement

The authors declare that the research was conducted in the absence of any commercial or financial relationships that could be construed as a potential conflict of interest.
